# Risk factors for and clinical implications of mixed *Candida*/bacterial bloodstream infections

**DOI:** 10.1111/j.1469-0691.2012.03906.x

**Published:** 2012-05-03

**Authors:** S-H Kim, Y K Yoon, M J Kim, J W Sohn

**Affiliations:** Division of Infectious Diseases, Department of Internal Medicine, Korea University College of MedicineSeoul, Korea

**Keywords:** Bacteraemia, candidaemia, co-infection, risk factors, treatment outcome

## Abstract

Mixed *Candida*/bacterial bloodstream infections (BSIs) have been reported to occur in more than 23% of all episodes of candidaemia. However, the clinical implications of mixed *Candida*/bacterial BSIs are not well known. We performed a retrospective case-control study of all consecutive patients with candidaemia over a 5-year period to determine the risk factors for and clinical outcomes of mixed *Candida*/bacterial BSIs (cases) compared with monomicrobial candidaemia (controls). Thirty-seven (29%) out of 126 patients with candidaemia met the criteria for cases. Coagulase-negative staphylococci were the predominant bacteria (23%) in cases. In multivariate analysis, duration of previous hospital stay ≥7 weeks (odds ratio (OR), 2.86; 95% confidence interval (CI), 1.09–7.53), prior antibiotic therapy ≥7 days (OR, 0.33; 95% CI, 0.14–0.82) and septic shock at the time of candidaemia (OR, 2.60; 95% CI, 1.14–5.93) were significantly associated with cases. Documented clearance of candidaemia within 3 days after initiation of antifungal therapy was less frequent in cases (63% vs. 84%; p = 0.035). The difference in the rate of treatment failure at 2 weeks was not significant between cases (68%) and controls (62%; p = 0.55). The crude mortality at 6 weeks and survival through 100 days did not differ between the two patient groups (p = 0.56 and p = 0.80, respectively). Mixed *Candida*/bacterial BSIs showed a lower clearance rate of candidaemia during the early period of antifungal therapy, although the treatment response and survival rate were similar regardless of concurrent bacteraemia. Further studies on the clinical relevance of species-specific *Candida*-bacterial interactions are needed.

## Introduction

*Candida* species are normal commensals of humans that commonly inhabit the gastrointestinal tract, the female genital tract and the skin, where diverse bacteria are most commonly found. *Candida* and bacteria directly and indirectly influence each other in many ways and can have important effects on each other’s survival, colonization and pathogenesis [1–3]. Although data on the clinical relevance of interactions between *Candida* and bacteria are still lacking, several studies have described associations between this genus of fungus and bacteria species in clinical specimens [4–6]. Previous studies have demonstrated both synergistic and antagonistic interactions between *Candida* and various bacteria species, including *Pseudomonas aeroginosa*, *Staphylococcus aureus* and *Acinetobacter baumannii* [7–11].

With the recent advances in medical technology, *Candida* species are the most common cause of invasive fungal infections. The occurrence of invasive candidiasis has increased over the past several decades, especially in immunocompromised or critically ill patients, and may contribute to high mortality and morbidity [12,13]. In most cases of candidaemia, the gastrointestinal tract and the skin have been considered the sources of infection [14]. As mentioned above, the extensive distribution of bacterial species can lead to candidaemia with concomitant bacteraemia. Mixed *Candida*/bacterial bloodstream infections (BSIs) have been reported to occur in >23% of all episodes of candidaemia [4]. However, the mechanisms of *Candida*-bacterial interaction in mixed *Candida*/bacterial BSIs and their clinical importance are not well known. Furthermore, very few studies have compared the clinical characteristics and outcomes of mixed *Candida*/bacterial BSIs and monomicrobial candidaemia [4,15]. Therefore, we conducted a retrospective, case-control study to determine the risk factors for and clinical implications of mixed *Candida*/bacterial BSIs.

## Methods

### Hospital setting

The retrospective, case-control study was conducted at Korea University Anam Hospital, a 950-bed tertiary care teaching hospital, located in Seoul, South Korea. There are three intensive care units (ICUs) of 56 beds. Blood cultures were processed by the automated system Vitek2 (bioMérieux, Marcy l’Étoile, France). This study was approved by the Institutional Review Board of the Korea University Anam Hospital. Informed consent was not required by the board because of the retrospective design of this study.

### Study design and definitions

We identified all patients aged ≥18 years who had at least one positive blood culture for *Candida* species and compatible clinical signs or symptoms from July 2006 to June 2011. Among these, mixed *Candida*/bacterial BSI cases were confirmed by isolation of bacterial species growing concomitantly from a single set or different sets of blood cultures obtained within a 48 h period. We used a standardized definition of contamination [16]. A blood culture was considered to be contaminated if one or more of the following organisms were identified only in one of a series of blood cultures: coagulase-negative staphylococci, *Propionibacterium acnes*, *Micrococcus* species, viridians group streptococci, *Corynebacterium* species or *Bacillus* species.

Medical records were reviewed to determine demographic data, co-morbidities and other risk factors associated with *Candida* infection within 30 days before positive blood cultures [17]. Neutropenia was defined as an absolute neutrophil count of <500 cells/mm^3^. Definitions of septic shock were adapted from the American College of Chest Physicians/Society of Critical Care Medicine Consensus Conference Committee [18]. Severity of illness at the onset of candidaemia was assessed using the sequential organ failure assessment (SOFA) score and the Pitt bacteraemia scoring system [19,20]. Source of infection was established by clinical evidence of infection regardless of whether causative organisms were recovered from the affected site. Catheter-related BSIs were defined according to the Infectious Diseases Society of America guidelines [21]. Early central venous catheter removal meant removing the central venous catheter within 48 h of drawing the first blood sample that was culture-positive for *Candida* species.

Blood cultures were usually obtained every 3 days until negative or when clinically indicated. The response to antifungal therapy at 2 weeks was evaluated by the guidelines for assessing treatment responses and defining study outcome in clinical trials of invasive fungal diseases published by the Mycoses Study Group and European Organisation for Research and Treatment of Cancer as follows: complete and partial responses were regarded as ‘success’ and stable response, progression of disease and death were regarded as ‘failure’ [22]. Candidaemia-attributable mortality was defined as any of the following: (i) blood cultures positive for *Candida* species at the time of death; (ii) death before the resolution of signs and symptoms related to candidaemia; or (iii) death at least 14 days after the onset of candidaemia without another explanation. The follow-up duration was 100 days after the first date of culture-positive blood samples for *Candida* species or until loss to follow-up or death from any cause. The parameters of mixed *Candida*/bacterial BSIs were compared with those of monomicrobial candidaemia.

### Statistical analysis

Continuous variables represented the median and interquartile range (IQR). The chi-squared test or Fisher’s exact test were used to compare categorical variables, and Student’s *t*-test was used to compare continuous variables. Variables with p-values of < 0.20 by univariate analysis were entered into the multivariable model. Multivariate analysis was performed by logistic regression analysis for the risk factors associated with mixed *Candida*/bacterial BSIs and Cox regression analysis for the predictive factors for mortality. Kaplan–Meier survival estimates were used to generate the survival curves and differences between survival curves were assessed by means of the log-rank test. Two-sided p-values of < 0.05 were considered statistically significant. Statistical analysis was performed using spss ver. 15.0 (SPSS Korea, Seoul, Korea).

## Results

### Patient characteristics

**FIG. 1:**
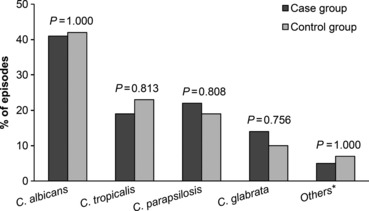
Comparison of the distribution of *Candida* species isolated from cases and controls. *Citrobacter famata (*n* = 2), *Citrobacter guilliermondii* (*n* = 2), *Citrobacter ciferrii* (*n* = 1), *Citrobacter krusei* (*n* = 1), *Citrobacter lusitaniae* (*n* = 1) and *Citrobacter sphaerica* (*n* = 1).

During a 5-year study period, we identified a total of 126 patients who had episodes of significant candidaemia in our institution. The overall incidence of candidaemia was 6.6 cases per 10 000 hospital admissions. The most commonly isolated *Candida* species was *Candida albicans* (41% of isolates), followed by *Candida tropicalis* (21%), *Candida parapsilosis* (20%) and *Candida glabrata* (11%). In no case was more than one *Candida* species isolated. Out of 126 patients, 37 (29%) showed mixed *Candida*/bacterial BSIs. The characteristics of the patients and the course of therapy in case and control patients are shown in Table 1. The two groups did not differ significantly in demographic characteristics, co-morbidities, risk factors for *Candida* infection or severity of illness. With regard to the source of candidaemia and antifungal therapy, there was no significant difference. The distribution of *Candida* species was similar in the two groups (Fig. 1).

**TABLE 1:** Demographic and clinical characteristics of the patients with mixed *Candida*/bacterial bloodstream infections (cases) compared with the patients with monomicrobial candidaemia (controls)

Characteristics	Cases (*n* = 37)	Controls (*n* = 89)	p
Age, median years (IQR)	63 (57–73)	68 (57–74)	0.15
Male sex	19 (51)	54 (61)	0.43
Co-morbidities			
Diabetes mellitus	10 (27)	24 (27)	1.00
Chronic kidney disease	6 (16)	10 (11)	0.56
Chronic liver disease	3 (8)	7 (8)	1.00
Solid tumour	17 (46)	43 (48)	0.85
Haematological disease	7 (19)	13 (15)	0.60
Charlson co-morbidity index, median (IQR)	2 (2–5.5)	2 (1–6)	0.78
Prior hospital stay, median days (IQR)	25 (11–60)	23 (11–38)	0.10
Risk factors for *Candida* infection^a^			
ICU residence	19 (51)	32 (36)	0.12
*Candida* colonization	7 (19)	13 (15)	0.60
Neutropenia	5 (14)	14 (16)	0.79
Chemotherapy	11 (30)	24 (27)	0.83
Corticosteroid^b^	12 (32)	15 (17)	0.06
Surgery	9 (24)	20 (23)	1.00
Hyperalimentation	29 (78)	79 (89)	0.16
Central venous catheter	30 (81)	65 (73)	0.38
Urinary catheter	26 (70)	56 (63)	0.54
Prior use of antibiotics	33 (89)	83 (93)	0.48
Prior azole exposure	5 (14)	11 (12)	1.00
Severity of illness			
SOFA score, median (IQR)	7 (3–10)	6 (2.5–9)	0.13
Pitt bacteraemia score, median (IQR)	4 (2–7.5)	3 (1–6)	0.14
Source of candidaemia			
Catheter	10 (27)	26 (29)	0.49
Gastrointestinal tract	13 (35)	41 (46)	
Others^c^	3 (8)	4 (5)	
Unknown	11 (30)	18 (20)	
First-line antifungal agent			
Fluconazole	18 (49)	45 (51)	0.63
Amphotericin B	9 (24)	15 (17)
Others^d^	2 (5)	3 (3)
None	8 (22)	26 (29)
Delay in initiation of antifungal therapy, median days (IQR)^e^	3 (1–4)	2 (1–3)	0.53
Total antifungal therapy, median days (IQR)	22 (7–27)	14 (7–21)	0.80

Data are no. (%) of patients, unless otherwise indicated.

ICU, intensive care unit; IQR, interquartile range; SOFA, sequential organ failure assessment.

a Risk factors within 30 days before candidaemia.

b A mean minimum dose of 10 mg/day of prednisone equivalent for >14 days.

c Genitourinary tract (*n* = 6) and deep soft tissue (*n* = 1).

d Itraconazole (*n* = 4) and caspofungin (*n* = 1).

e The time between drawing the first blood samples culture-positive for *Candida* species and initiation of antifungal therapy.

**FIG. 2:**
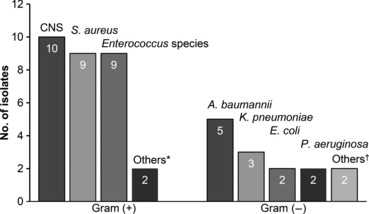
Distribution of the bacterial species isolated from 37 cases. CNS, coagulase-negative staphylococci. *Leuconostoc pseudomesenteroides (*n* = 1) and Gram-positive bacillus (*n* = 1). ^†^*Citrobacter freundii* (*n* = 1) and *Brevundimonas vesicularis* (*n* = 1).

Various bacterial organisms, a total of 44 isolates, were isolated from 37 patients (Fig. 2). Five patients had two and one patient had three bacterial species isolated. Gram-positive organisms accounted for more than 68% (30/44) of all bacterial isolates. Coagulase-negative staphylococci (23%, 10/44) were the most prevalent bacterial pathogen. The central venous catheter (30%, 11/37) and the gastrointestinal tract (30%, 11/37) were the most common sources of bacteraemia, followed by the lower respiratory tract (19%, 7/37), unknown sources (16%, 6/37) and the urinary tract (5%, 2/37). Appropriate antibiotics were administered to all cases except three (one for methicillin-resistant coagulase-negative staphylococcus and two for multidrug-resistant *Acinetobacter baumannii*). Appropriate antibiotic therapy was started on a median of 1 day (IQR, 0–11 days) after the onset of bacteraemia.

### Risk factors for mixed *Candida*/bacterial BSIs

In univariate analysis, cases tended to have had a previous hospital stay of ≥7 weeks (30% vs. 16%; p = 0.08) and to have received corticosteroids prior to the onset of candidaemia, compared with controls (32% vs. 17%; p = 0.06). Prior antibiotic use for ≥7 days was associated with a lower chance of having mixed *Candida*/bacterial BSIs (62% vs. 80%; p = 0.04). Cases more commonly manifested as septic shock at the time of candidaemia (51% vs. 32%; p = 0.04). Table 2 shows the results of univariate and multivariate analyses of risk factors for mixed *Candida*/bacterial BSIs. After multivariate analysis, previous hospital stay for ≥7 weeks, prior antibiotic use for ≥7 days and the presence of septic shock at the time of candidaemia were significantly associated with mixed *Candida*/bacterial BSIs.

**TABLE 2:** Logistic regression analysis for risk factors associated with mixed *Candida*/bacterial bloodstream infections

Characteristics	OR (95% CI)	p	Adjusted OR (95% CI)	p
Duration of previous hospital stay ≥7 weeks	2.27 (0.92–5.61)	0.08	2.86 (1.09–7.53)	0.033
ICU residence	1.88 (0.87–4.09)	0.11	–	0.24
Corticosteroid^a^	2.37 (0.98–5.73)	0.06	–	0.10
Hyperalimentation^a^	0.46 (0.17–1.28)	0.14	–	0.16
Duration of prior antibiotic therapy ≥7 days^a^	0.42 (0.18–0.97)	0.04	0.33 (0.14–0.82)	0.016
Septic shock	2.30 (1.05–5.04)	0.04	2.60 (1.14–5.93)	0.024
SOFA score ≥10	1.87 (0.81–4.32)	0.14	–	0.42
Pitt bacteraemia score ≥8	2.06 (0.79–5.42)	0.14	–	0.41

ICU, intensive care unit; OR, odds ratio; SOFA, sequential organ failure assessment.

a Risk factors within 30 days before candidaemia.

### Clinical outcome of mixed *Candida*/bacterial BSIs

The clinical outcomes of cases and controls are shown in Table 3. Within 3 days after initiation of antifungal therapy, documented clearance of candidaemia was significantly infrequent in cases (p = 0.035; Table 3). After adjustment for the first-line antifungal agent, ≥48 h delay in initiation of antifungal therapy and early removal of central venous catheter by multivariate analysis, mixed *Candida*/bacterial BSI was only a significant factor associated with clearance of candidaemia at day 3 (OR, 0.26; 98% CI, 0.08–0.84; p = 0.024). For the treatment response at 2 weeks, 25 of 37 (68%) cases and 55 of 89 (62%) controls had a treatment failure (p = 0.55). Of the reasons for treatment failure, microbiological failure was observed for 14% of cases and 6% of controls (p = 0.16). In subgroup analysis according to *Candida* and bacterial species, there were no significant differences in the documented clearance of candidaemia within 3 days or treatment response at 2 weeks (data not shown).

**TABLE 3:** Comparison of clinical outcomes between patients with mixed *Candida*/bacterial bloodstream infections (cases) and those with monomicrobial candidaemia (controls)

Variables	Cases (*n* = 37)	Controls (*n* = 89)	p
Documented clearance of candidaemia within 3 days after initiation of antifungal therapy^a^	19/30 (63)	48/57 (84)	0.035
Treatment failure at 2 weeks	25 (68)	55 (62)	0.55
Clinical failure^b^	5 (14)	13 (15)	1.00
Microbiological failure^c^	5 (14)	5 (6)	0.16
Death	15 (41)	37 (42)	1.00
Mortality at 6 weeks			
Crude	24 (65)	50 (56)	0.43
Candidaemia-attributable	16 (43)	37 (42)	1.00

Data are no. (%) of patients, unless otherwise indicated.

a Data are event/evaluable no. (%) of patients.

b Worsening or no improvement of attributable symptoms or signs of candidaemia.

c Persistent isolation of *Candida* species from blood specimens at 2 weeks.

**FIG. 3:**
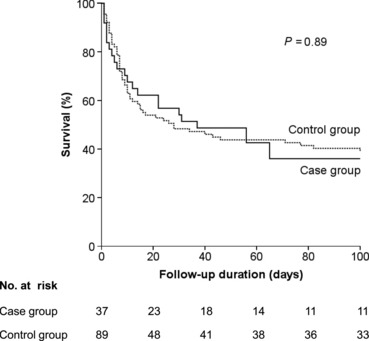
Kaplan–Meier estimates of survival in patients with mixed *Candida*/bacterial bloodstream infection and monomicrobial candidaemia.

Overall mortality at 6 weeks was 59% (74/126) of all candidaemic patients. Thirty-four (27%) patients did not receive any antifungal therapy; of these, 28 (82%) died at week 6. Differences between cases and controls were not significant in the crude and candidaemia-attributable mortality at 6 weeks (p = 0.43 and p = 1.00, respectively; Table 3). In univariate analysis, mixed *Candida*/bacterial BSI was not predictive of crude mortality at 6 weeks (p = 0.72). Predictive factors of the 6-week mortality are shown in Table 4. After multivariate analysis, a Charlson co-morbidity index ≥4, surgery, SOFA score ≥10, Pitt bacteraemia score ≥8, delay in initiation of antifungal therapy >72 h and persistent candidaemia at the last blood culture remained independent predictors for crude mortality at 6 weeks. Kaplan–Meier estimates of survival are shown in Fig. 3. The median time to death was 45 days in cases and 40 days in controls (p = 0.89).

**TABLE 4:** Predictors of the 6-week mortality in all candidaemic patients

Characteristics	HR (95% CI)	p	Adjusted HR (95% CI)	p
Mixed *Candida*/bacterial BSI	1.09 (0.67–1.78)	0.72	–	ND
Gram-positive bacteria	0.76 (0.42–1.39)	0.37	–	ND
Gram-negative bacteria	1.67 (0.86–3.25)	0.13	–	0.15
Co-morbidities				
Chronic kidney disease	2.03 (1.11–3.72)	0.021	–	0.96
Charlson co-morbidity index ≥4	1.79 (1.13–2.85)	0.014	1.91 (1.17–3.13)	0.01
Risk factors for *Candida* infection^a^				
ICU residence	1.77 (1.12–2.80)	0.015	–	0.36
Neutropenia	1.46 (0.82–2.62)	0.20	–	0.08
Chemotherapy	1.56 (0.95–2.54)	0.08	–	0.15
Corticosteroids	1.59 (0.94–2.68)	0.09	–	0.27
Surgery	0.46 (0.24–0.88)	0.02	0.34 (0.17–0.65)	0.001
Hyperalimentation	1.72 (0.83–3.60)	0.15	-	0.31
Severity of illness				
SOFA score ≥10	3.78 (2.33–6.12)	<0.001	3.00 (1.73–5.18)	<0.001
Pitt bacteraemia score ≥8	4.83 (2.80–8.33)	<0.001	6.98 (3.32–14.70)	<0.001
Mechanical ventilation	1.90 (1.19–3.02)	0.007	–	0.76
Continuous renal replacement therapy	1.90 (1.02–3.54)	0.04	–	0.07
Delay in initiation of antifungal therapy >72 h	1.87 (1.16–3.00)	0.01	2.03 (1.23–3.35)	0.006
Early central venous catheter removal	0.68 (0.41–1.13)	0.13	–	0.99
Persistent candidaemia at the last blood culture	5.87 (3.62–9.52)	<0.001	7.60 (4.36–13.25)	<0.001

BSI, bloodstream infection; HR, hazard ratio; ICU, intensive care unit; SOFA, sequential organ failure assessment.

a Risk factors within 30 days prior to candidaemia.

## Discussion

In this study, a significant portion of candidaemia episodes occurred concurrently with bacteraemia. Compared with monomicrobial candidaemia, mixed *Candida*/bacterial BSIs were associated with a prolonged length of previous hospital stay (≥7 weeks) and septic shock at the time of candidaemia. Prior receipt of antibiotic agents for ≥7 days was related to monomicrobial candidaemia rather than mixed *Candida*/bacterial BSIs. We found that documented clearance of candidaemia during the early period of antifungal therapy was deferred in patients with concurrent bacteraemia. However, treatment responses at 2 and 6 weeks and survival through 100 days were not significantly different between mixed *Candida*/bacterial BSIs and monomicrobial candidaemia.

Mixed *Candida*/bacterial BSIs have been commented in several articles on candidaemia, the results of which have suggested that the incidence of concurrent bacteraemia accounts for 23% of all episodes of candidaemia [4,23]. Thorn *et al.* [24] reported that 39% of the post-mortem blood cultures with *Candida* species were polymicrobial. We observed a similar incidence in previous studies. However, the concept of ‘polymicrobial BSI’ or ‘concomitant BSI’ was not properly identified in most previous articles and an official definition has still not been established. The following definition has mainly been used: the isolation of bacterial species synchronously with *Candida* species or within 48 h of the time of candidaemia. Previous studies have reported the incidence of concurrent bacteraemia to be 7–27%. Abi-Said *et al.* [25] showed that concomitant bacterial infection defined as isolation of bacteria 1 week prior to or after the time of candidaemia occurred in 57% of patients. In this study, we used the term ‘mixed *Candida*/bacterial BSI’ to describe the isolation of bacterial species within 48 h of the time of candidaemia to avoid confusion with previous studies. We chose this definition because concomitant bacteraemia may obscure the detection of fungaemia using standard blood culture techniques by suppression of fungal growth [9,10,26]. The inclusion of only synchronous candidaemia and bacteraemia could cause the underestimation of the incidence of mixed *Candida*/bacterial BSIs.

We found that clearance of candidaemia within 3 days after the start of antifungal therapy was significantly infrequent in mixed *Candida*/bacterial BSIs, compared with monomicrobial candidaemia. This result might be explained by the following mechanisms: the synergistic relationship between *Candida* and bacteria species enhances the viability of *Candida* [27,28], and/or fungal growth is increased by antibacterial treatment for co-infected bacteria with an antagonistic effect on *Candida* species [10,29]. However, the clinical significance of synergism between *Candida* and bacteria species has not been proven, and quantitative blood culture was not performed in this study. Further *in vitro* and *in vivo* investigations are needed to test this hypothesis.

It is not yet clear whether mixed-species infections have different clinical outcomes than single-species infections. Several studies have reported significant implications of *Candida*-bacterial interactions in animal models and various clinical settings, including biofilms on implanted medical devices, peritonitis and ventilator-associated pneumonia [6,8,30,31]. As far as we know, only one study has compared clinical outcomes in BSIs by mixed *Candida*-bacteria species and *Candida* species alone. Dyess *et al.* [15] reported a poorer clinical outcome in patients with synchronous candidaemia and bacteraemia (39% survival) than in patients with only candidaemia (67% survival; p < 0.05). However, they did not present the demographic characteristics, underlying disease and severity of illness in each patient group. Unlike their result, our study showed no difference in clinical outcome between mixed *Candida*/bacterial BSIs and monomicrobial candidaemia. Concomitant bacteraemia was also not a predictive factor of the 6-week mortality. Mortality at 6 weeks was associated with the Charlson co-morbidity index, SOFA score, Pitt bacteraemia score, recent surgery, delayed start of antifungal therapy and persistent candidaemia. These findings suggest that chronic co-morbid conditions and severity of illness at the onset of candidaemia are the key factors of clinical outcome of candidaemic patients.

This study has several limitations. Because we performed a retrospective analysis with single-centre data, the distribution of the causative pathogens (both *Candida* and bacteria species) in our institution could have had an impact on the results. Also, a relatively small number of cases caused by each bacterial species was included and we did not analyse the clinical influence of mixed *Candida*/bacterial BSIs according to the bacterial species. However, to the best of our knowledge, this is the first report on the risk factors for and clinical outcomes of mixed *Candida*/bacterial BSIs compared with monomicrobial candidaemia. Our results suggest that empirical antibacterial and antifungal therapy should be properly considered on the basis of local microbiological epidemiology and susceptibility profiles in cases with risk factors for mixed *Candida*/bacterial BSIs. Although mixed *Candida*/bacterial BSIs are not predictive for a poor prognosis with appropriate antimicrobial therapy, more aggressive policies to clear *Candida* species from blood might be required in the early period of antimicrobial therapy to minimize the possibility of metastatic complications.

In our study, mixed *Candida*/bacterial BSIs accounted for a considerable part of candidaemia. We found several risk factors for and clinical implications of mixed *Candida*/bacterial BSIs. The interactions between *Candida* and bacteria species are highly complex and their clinical impact could be influenced by host factors, microbial pathogen and antimicrobial therapy. To understand the mechanisms of *Candida*-bacterial interactions in the bloodstream and exploit it for therapeutic strategies, further studies using *in vitro* or animal model systems as well as large-scale, multicentre clinical data are needed.
